# Hypoxia-Derived Exosomes Promote Lung Adenocarcinoma by Regulating HS3ST1-GPC4-Mediated Glycolysis

**DOI:** 10.3390/cancers16040695

**Published:** 2024-02-06

**Authors:** Xianxiu Ji, Ren Zhu, Caixia Gao, Huikang Xie, Xiaomei Gong, Jie Luo

**Affiliations:** 1 Department of Oncology, Shanghai Pulmonary Hospital, School of Medicine, Tongji University, Shanghai 200433, China; jixianxiu@163.com; 2 Department of Medical Administration, Shanghai Pulmonary Hospital, School of Medicine, Tongji University, Shanghai 200433, China; 3 Department of Pathology, Shanghai Pulmonary Hospital, School of Medicine, Tongji University, Shanghai 200433, China; 4 Department of Radiation Oncology, Shanghai Pulmonary Hospital, School of Medicine, Tongji University, Shanghai 200433, China

**Keywords:** lung adenocarcinoma (LUAD), lncRNA, glycolysis, miRNA, HS3ST1, GPC4

## Abstract

**Simple Summary:**

Lung cancer accounts for 11.4% of all cancer cases and 18.0% of all cancer deaths. Understanding the molecular mechanisms underlying lung adenocarcinoma (LUAD), one of the most common lung cancers, is imperative to enhance diagnostic accuracy, drug development, and prognostic evaluation. The present study explored the interaction between heparan sulfate (glucosamine) 3-O-sulfotransferase 1 (HS3ST1) and Glypican 4 (GPC4), as well as the influence of the hypoxia-derived exosomal lncRNA OIP5-AS1 on the glycolysis, proliferation, and metastasis ability in LUAD cell lines and their effects on the tumor growth in xenograft animal models. GPC4 promotes HS3ST1-mediated glycolysis, and the hypoxia-derived exosomal lncRNA OIP5-AS1 promotes glycolysis via the miR-200c-3p axis in LUAD cells. The hypoxia-derived exosomal lncRNA OIP5-AS1 enhances LUAD cell proliferation and metastasis in vitro and promotes LUAD tumor growth and metastasis via miR-200c-3p in vivo. These findings highlight that the hypoxia-derived exosomal lncRNA OIP5-AS1 may participate in LUAD progression.

**Abstract:**

Objective: The diagnosis of lung adenocarcinoma (LUAD) is often delayed due to the typically asymptomatic nature of the early-stage disease, causing advanced-stage LUAD diagnosis in most patients. Hypoxia is widely recognized as a driving force in cancer progression. Exosomes originating from hypoxic tumor cells promote tumorigenesis by influencing glycolysis, migration, invasion, and immune infiltration. Given these insights, our study aimed to explore the role of hypoxia-derived exosomal long non-coding RNA (lncRNA) OIP5-AS1 in LUAD cell lines and mouse models. Materials and Methods: Exosomes were meticulously isolated and authenticated based on their morphology and biomarkers. The interaction between heparan sulfate (glucosamine) 3-O-sulfotransferase 1 (HS3ST1) and Glypican 4 (GPC4) was examined using immunoprecipitation. The influence of the hypoxia-derived exosomal lncRNA OIP5-AS1 on glycolysis was assessed in LUAD cell lines. The effect of the hypoxia-derived exosomal lncRNA OIP5-AS1 on cell proliferation and metastasis was evaluated using colony formation, cell viability, cell cycle, and apoptosis analyses. Its effects on tumor size were confirmed in xenograft animal models. Results: Our study revealed the mechanism of the hypoxia-derived exosomal lncRNA OIP5-AS1 in LUAD progression. We discovered that GPC4 promotes HS3ST1-mediated glycolysis and that the hypoxia-derived exosomal lncRNA OIP5-AS1 enhances glycolysis by regulating miR-200c-3p in LUAD cells. Notably, this lncRNA stimulates LUAD cell proliferation and metastasis and fosters LUAD tumor size via miR-200c-3p. Our findings underscore the potential role of the hypoxia-derived exosomal lncRNA OIP5-AS1 in LUAD progression. Conclusions: The hypoxia-derived exosomal lncRNA OIP5-AS1 promotes LUAD by regulating HS3ST1-GPC4-mediated glycolysis via miR-200c-3p.

## 1. Introduction

Lung cancer is the second most frequently identified cancer type, accounting for 11.4% of all cancer cases. It is also the foremost contributor to global cancer-related fatalities, accounting for 18.0% of all cancer deaths [1]. One of the most common lung cancers is lung adenocarcinoma (LUAD), whose early diagnosis is frequently delayed due to the absence of early-stage symptoms, ultimately causing advanced-stage diagnoses for most patients with the disease. Alarmingly, only 30% of patients are diagnosed with stage I cancer, yielding a 5-year survival rate of 65%, which precipitously declines to 5% or 6% in advanced patients [2,3]. Consequently, understanding the molecular mechanisms underlying LUAD incidence and progression is imperative for enhancing diagnostic accuracy, drug development, and prognostic evaluation.

Hypoxia is widely recognized as a crucial driver of cancer progression [4,5]. It triggers adaptive responses involving stromal cell alterations within the tumor microenvironment, supporting cancer cell survival, development, and aggressiveness [4,6]. Diminished oxygen levels activate the SET domain-containing 2 (HIF-1) protein, a transcription factor composed of HIF-1α and HIF-1β subunits [7]. Under hypoxic conditions, the HIF-1α subunit stabilizes and accumulates along with other hypoxia-linked proteins, correlating with unfavorable outcomes in individuals with lung cancer [8]. In contrast, under normal oxygen conditions, the HIF-1α subunit destabilizes and degrades, indicating that it indirectly affects normoxic cancer cells.

Exosomes are tiny, 30–100 nm, single-membrane vesicles that are actively secreted by numerous cell types, including tumor cells [9]. They serve as carriers for different nucleic acids, such as circular RNAs, long non-coding RNAs (lncRNAs), and microRNAs (miRNAs) [10]. Hypoxia can accelerate tumorigenesis by boosting the release of exosomes and altering their contents [11]. Exosomes derived from hypoxic tumor cells fuel tumor progression by influencing glycolysis, cell migration, invasion, and immune infiltration. Thus, they are potential carriers of hypoxic signals that directly affect normoxic cancer cells [12,13].

Transcripts exceeding 220 nucleotides in length are called lncRNAs and play essential roles in diverse cellular processes such as cell cycle regulation, apoptosis, and genome maintenance [14,15,16,17]. These aberrations are closely associated with cancer development, progression, metastasis, and drug resistance [18,19,20,21]. For instance, long intergenic non-protein coding RNA 968 (LINC00968) acts as an oncogenic factor in non-small-cell lung cancer (NSCLC) via the WNT signaling pathway [22]. Differentiation antagonizing non-protein coding RNA (DANCR) is a competing endogenous RNA for microRNA 496 (miR-496) that influences the mechanistic target of rapamycin kinase (mTOR) protein expression and promotes NSCLC progression [23]. Furthermore, long intergenic non-protein coding RNA 473 (LINC00473) is upregulated by the inactivation of serine/threonine kinase 11 (LKB1), enhancing the proliferation of LKB1-inactivated lung cancer cells [24]. Together, these findings highlight the pivotal role of lncRNAs in lung cancer pathogenesis and their potential utility as novel diagnostic and therapeutic biomarkers.

In this study, we investigated the influence of hypoxia-derived exosomes on LUAD progression. We found that glypican 4 (GPC4) promoted heparan sulfate-glucosamine 3-sulfotransferase 1 (HS3ST1)-mediated glycolysis in LUAD cells. Moreover, these cells expressed the exosomal lncRNA OIP5 antisense RNA 1 (OIP5-AS1), which enhances glycolysis by regulating miR-200c-3p during hypoxia, boosting cancer cell proliferation, tumor growth, and metastasis. Our findings highlight that the hypoxia-derived exosomal lncRNA OIP5-AS1 may participate in LUAD progression.

## 2. Materials and Methods

### 2.1. Gene Expression Profiling Interactive Analysis (GEPIA)

The TCGA data analysis was conducted using the GEPIA website (http://gepia.cancer-pku.cn/ accessed on 23 November 2022) as previously described [25]. Firstly, the levels of HS3ST1 and GPC4 were analyzed in 31 kinds of tumor tissues and corresponding normal tissues. Next, the levels of HS3ST1 and GPC4 in LUAD tissue and normal tissue were analyzed, respectively. The influence of HS3ST1 expression on patient survival probability were then analyzed.

### 2.2. Cell Culture

The lung adenocarcinoma cell lines H1560 and A-549 were obtained and cultured in T-75 flasks with Dulbecco’s modified Eagle’s medium (HyClone Laboratories, Logan, UT, USA) supplemented with 10% fetal bovine serum (Beyotime Biotechnology, Shanghai, China). The cells were maintained at 37 °C in a 5% CO_2_ environment, with medium replacement every 2 days.

### 2.3. Co-Immunoprecipitation (Co-IP)

All IP experiments were performed using A-549 cells. Briefly, A-549 cells were lysed with ice-cold lysis buffer (150 mM of NaCl, 10 mM of Tris–HCl (pH 7.5), 0.5 mM of EDTA, 0.5% NP-40), then centrifuged at 15,000× *g* for 15 min at 4 °C. Next, the supernatant was collected into a new tube. A total of 5 μg of an immunoprecipitation antibody (Sigma-Aldrich, St. Louis, MO, USA) were added into the tube with the lysate, and the tube was incubated on a rotator for 1 h at 4 °C. Then, 20 μL of resin was added to each reaction tube and incubated on a rotator for 1 h at 4 °C. Finally, the resin-bound immune complexes were resuspended in 25 μL of 2 × Laemmli buffer, boiled for 10 min, and analyzed by Western blotting. The uncropped blots and molecular weight markers are shown in Supplementary Figure S1.

### 2.4. Immunofluorescence Staining

Briefly, A-549 cells (1 × 10^4^) were seeded on coverslips in 0.5% FBS and allowed to adhere. They were then incubated with the first primary antibodies (anti-HS3ST1, anti-GPC4, Sigma-Aldrich, USA) in 1% BSA overnight at 4 °C, washed with PBS, and incubated with the corresponding secondary antibody (Sigma-Aldrich, USA) in 1% BSA for 1 h at 25 °C. Next, the cells were counterstained with the nuclear stain DAPI (I Sigma-Aldrich, USA) as indicated.

### 2.5. Mitochondrial Stress Test

To evaluate mitochondrial stress, the oxygen consumption rate (OCR) was measured using a Cell Mito Stress Test kit (Agilent Technologies, Inc., Santa Clara, CA, USA) [26]. Briefly, 96-well microplates were seeded with A-549 cells at approximately 1 × 10^4^ cells/well. The cells were incubated in a non-buffered assay medium for 60 min in a non-CO_2_ incubator. The recording commenced by establishing baseline OCR values, followed by sequential injections of 1 μM of oligomycin, 1 μM of carbonyl cyanide-4 (trifluoromethoxy) phenylhydrazone (FCCP), 1 μM of rotenone, and 20 μM of antimycin A. 

### 2.6. Glycolysis Stress Test

The extracellular acidification rate (ECAR) was measured using an XF Cell Glycolysis Test Kit (Agilent Technologies, Inc., USA). Briefly, approximately 5 × 10^4^ A-549 cells per well were seeded onto XF24 microplates and incubated for 60 min in a non-buffered assay medium without CO_2_. The recording began by establishing baseline ECAR values followed by sequential injections of 10mM of glucose, 1 μM of oligomycin, or 50 mM of 2-deoxy-D-glucose (2-DG).

### 2.7. Glucose Uptake and Lactate Product Assay

Glucose uptake was assessed by quantifying the glucose concentration in the medium (Sigma, USA), and lactate production was evaluated using a Lactate Assay Kit (Solarbio, Beijing, China), following the manufacturer’s instructions. Before the assessments, all metabolic parameters were standardized based on the total cell count.

### 2.8. Western Blotting Assay

Total protein was extracted from the cultured cells using the RIPA buffer (Beyotime Biotechnology, China). The protein quantity and concentration in the extract were determined using a bicinchoninic acid assay (Beyotime Biotechnology, China). Protein extracts were diluted and loaded onto a 12% polyacrylamide gel (Bio-Rad Laboratories Inc., Hercules, CA, USA). The proteins were separated by electrophoresis and transferred onto a 0.45 μm polyvinylidene fluoride membrane (GE HealthCare, Chicago, IL, USA). The membranes were blocked with non-fat dry milk (Beyotime Biotechnology, China) and incubated with primary antibodies at 4 °C overnight. A 2 h incubation with secondary antibodies was performed at room temperature. Images of the membranes were obtained using a chemiluminescent HRP substrate (Millipore, Burlington, MA, USA) in a ChemiDoc MP imaging system (Bio-Rad Laboratories, USA). The glyceraldehyde-3-phosphate dehydrogenase (GAPDH) was used as the control. All Western blot antibodies are listed in Supplementary Table S1.

### 2.9. Exosome Isolation and Detection

Exosomes were collected from normoxic or hypoxic LUAD A-549 cells using the ExoQuick precipitation kit (System Biosciences, Inc., Palo Alto, CA, USA). Briefly, for normoxic LUAD A-549 cells, the cells were cultured under 20% O_2_ in exosome-free DMEM for 48 h; for hypoxic LUAD A-549 cells, the cells were cultured under 1% O_2_ in exosome-free DMEM for 48 h. After collecting the conditioned medium, the exosomes were extracted following instruction. Their morphology was examined using transmission electron microscopy (TEM), and their size distribution was assessed using nanoparticle tracking analysis (NTA). Exosomal markers were confirmed by Western blotting.

### 2.10. Cell Transfection

A-549 cells were transfected with small interfering RNAs (siRNAs) or short hairpin RNAs (shRNAs) using a Lipofectamine transfection reagent (Beyotime Biotechnologies). Following a 6 h transfection period, the cells were moved to a complete medium and cultivated for an additional 30 h.

### 2.11. Reverse Transcription-Quantitative PCR (RT-qPCR)

Total RNA was extracted from the cells using the TRIzol reagent (Life Technologies, Carlsbad, CA, USA) and reverse-transcribed to cDNA using a cDNA synthesis kit (Vazyme, San Diego, CA, USA) following the manufacturer’s guidelines. A quantitative PCR was conducted using the SsoFast EvaGreen Supermix Kit (Bio-Rad, USA). All relative expression data were normalized to the GAPDH endogenous control. The primers used in this study are listed in Supplementary Table S2.

### 2.12. Cell Viability Analysis

The cell viability was assessed using the cell counting kit-8 (CCK-8) (Abcam, Cambridge, UK). Lung adenocarcinoma A-549 cells were plated in 96-well dishes at 6 × 10^3^ cells per well and incubated for 1 d before treatment. The CCK-8 solution was added to each well, followed by a 3 h incubation period. Absorbance at 450 nm was measured using a microplate reader (PerkinElmer, Inc., Shelton, CT, USA).

### 2.13. Colony Formation Assays

The cells were cultivated in 6-well dishes at a seeding density of 1 × 10^3^ cells/well, and the culture medium was replaced every 48 h. Following a 10-day incubation, the colonies were fixed and treated with crystal violet. The number of colonies was then quantified.

### 2.14. Cell Cycle and Apoptosis Analyses

A cell cycle analysis was conducted 24 h post-transfection using a cell cycle detection kit (Abcam, Cambridge, UK). Following overnight fixation in 95% ethanol, the cells were stained with 50 μg/mL of propidium iodide (PI) in the dark for 30 min. Flow cytometry was used to determine the distribution of cells across the various cell cycle stages.

For apoptosis assessment, the transfected cells were enzymatically dissociated and stained with an Annexin V-FITC apoptosis detection kit (Abcam, UK). The cell apoptosis rate was quantified using flow cytometry.

### 2.15. Dual-Luciferase Reporter Assay

A dual-luciferase reporter assay was conducted according to the manufacturer’s instructions using an appropriate kit (Beyotime Biotechnology, China). Four different promoter sequences (350, 500, 1000, and 6000 bps) of the *HS3ST1* gene were amplified and integrated into the pGL3 reporter vector. Human 293T cells were cultivated in a 12-well dish and temporarily transfected with plasmids bearing different promoters using Lipofectamine 2000 (Thermo Fisher Scientific Inc., USA). For each transfection, 1 μg of the reporter plasmid and 1 μg of Renilla luciferase were used. After 48 h, the cells were lysed using PLB buffer and the luciferase signals were quantified.

### 2.16. Xenograft Tumor Experiments

Twenty-four 4-week-old BALB/c nude mice weighing 21–25 g were obtained from Shanghai MODEL ORGANISMS (Shanghai, China) and maintained under specific pathogen-free conditions in accordance with NIH guidelines. The animals had continuous access to sterile rodent chow and water and were housed in sterile filter-top cages under a 12 h light/dark cycle. Human lung adenocarcinoma A-549 cells (2 × 10^6^ cells per mouse) were subcutaneously administered to the mice, and the tumor dimensions were regularly assessed every 3 days. The tumor volume was determined using the following formula: V(cm^3^) = 1/2 × length × width^2^. After 8 weeks, xenograft tumors were collected and stained with hematoxylin–eosin staining. The Laboratory Animal Management Committee of Shanghai Pulmonary Hospital approved the animal study protocol.

### 2.17. Samples Collection

Thirty-six pairs of tumor tissues and non-tumor tissues (greater than 5 cm from tumor tissue) were collected from LUAD patients admitted to Shanghai Pulmonary Hospital from March 2017 to April 2021. Patients with primary LUAD who had not received chemotherapy or other treatments before surgery were included. Tissue specimens were histopathologically and clinically diagnosed and stored at −80 °C. Moreover, serum samples were collected from thirty-one healthy control individuals, pneumonia patients, and NSCLC patients admitted to Shanghai Pulmonary Hospital from March 2017 to April 2021 to measure the levels of the lncRNA OIP5-AS1. This study was approved by the Ethics Committee of Shanghai Pulmonary Hospital, and written informed consent was obtained from all participants.

## 3. Statistical Analysis

Statistical analyses were performed using SPSS Statistics for Windows (version 17.0; SPSS, Inc., Chicago, IL, USA). Quantitative data were analyzed using a one-way analysis of variance (ANOVA) with Dunnett’s post hoc test for multiple comparisons to determine statistical significance. The results are presented as mean ± standard deviation, with significance set at *p* < 0.05.

## 4. Results

*HS3ST1* and *GPC4* are overexpressed and interact in LUAD tissues.

**Figure 1:**
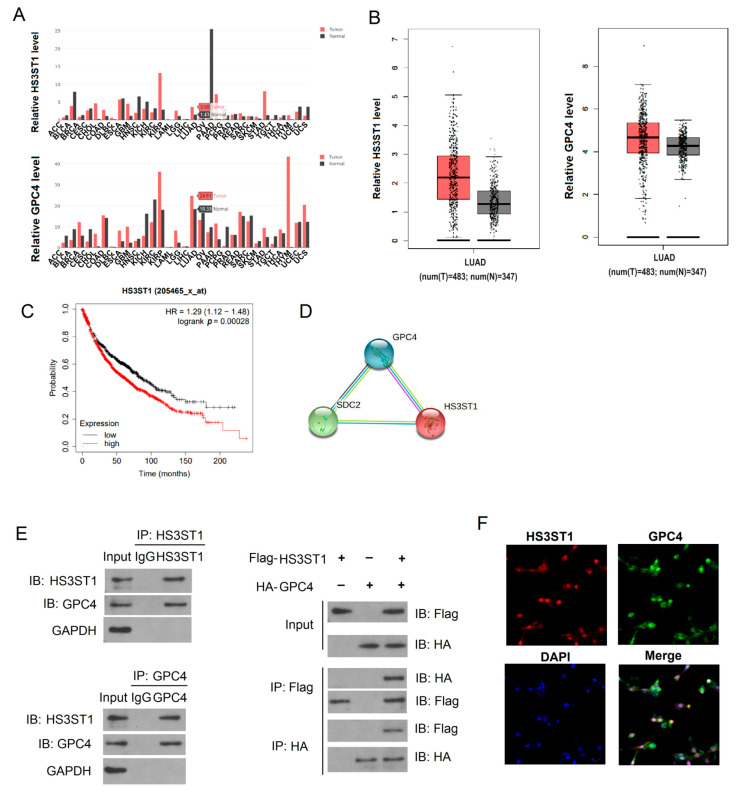
HS3ST1 is overexpressed and interacts with GPC4 in LUAD tissues: (**A**) GEPIA dataset showing *HS3ST1* or GPC4 mRNA expression in 31 neoplasm types. (**B**) GEPIA dataset with *HS3ST1* or *GPC4* mRNA expression in LUAD tissue samples (n = 483) and controls (n = 347). (**B**) The lung injury score of lung tissues in IR mice after salidroside or Fer-1 treatment. (**C**) LUAD prognosis in patients with low or high *HS3ST1* expression. (**D**) Bioinformatic analysis revealing the relationship between HS3ST1 and GPC4 proteins. (**E**) Immunoprecipitation assay confirming direct interaction between HS3ST1 and GPC4. (**F**) Immunofluorescence of HS3ST1 and GPC4 in LUAD tissues.

We retrieved RNA sequencing data from the GEPIA dataset, containing 483 LUAD and 347 control samples. Interestingly, we observed that *HS3ST1* and *GPC4* were significantly overexpressed in LUAD tissues (Figure 1B). Additionally, our analysis of RNA sequencing data across 31 types of neoplasms revealed that these two genes are upregulated in many cancers, including LUAD (Figure 1A). Moreover, HS3ST1 expression was positively associated with poor prognosis in patients with LUAD (Figure 1C).

Subsequently, we performed a bioinformatics analysis to clarify the relationship between HS3ST1 and GPC4, which suggested a potential association between the two proteins in LUAD tissues (Figure 1D). We conducted a co-IP assay using specific antibodies against HS3ST1 or GPC4 to verify whether HS3ST1 physically interacted with GPC4 in LUAD tissues. HS3ST1 co-immunoprecipitated with GPC4, confirming a tangible interaction between HS3ST1 and GPC4 (Figure 1E). Moreover, immunofluorescence demonstrated that HS3ST1 colocalized with GPC4 in the nucleus of LUAD cells, indicating that they regulate target genes in the nucleus of LUAD cells (Figure 1F). In conclusion, these findings show HS3ST1 and GPC4 overexpression and nuclear interactions in LUAD tissues.

### 4.1. HS3ST1 Promotes Glycolysis in LUAD Cells

**Figure 2:**
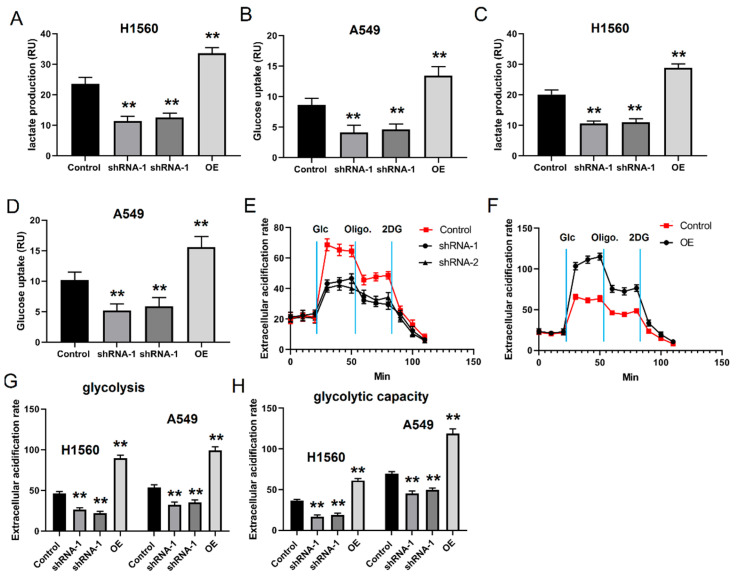
HS3ST1 promotes glycolysis in LUAD cells: (**A**,**C**) Lactate production of LUAD H1560 or A-549 cells after knocking down or overexpressing *HS3ST1*. (**B**,**D**) Glucose uptake of LUAD H1560 or A-549 cells after knocking down or overexpressing *HS3ST1*. (**E**–**H**) The ECAR of LUAD H1560 or A-549 cells after knocking down or overexpressing *HS3ST1*. GAPDH was used for normalization. Error bars represent the means ± SEM of 3 independent biological experiments. ** *p* < 0.01. ECAR denotes extracellular acidification rate.

Even when oxygen is sufficient, cancer cells exhibit an inclination towards glycolysis as their preferred pathway for glucose metabolism, a phenomenon known as the Warburg effect [27]. We selected H1560 and A-549 human LUAD cells to investigate the role of HS3ST1 in LUAD pathogenesis. We perturbed *HS3ST1* expression in these cells by overexpressing or knocking down the gene. Glucose uptake and lactate assays are always used to evaluate the level of glycolysis in vitro. Our research found that knocking down *HS3ST1* in the cells caused a significant decrease in lactate production and glucose uptake, implying that decreased *HS3ST1* expression downregulates glycolysis in LUAD cells (Figure 2A–D). Conversely, *HS3ST1* overexpression significantly increased the lactate production and glucose uptake (Figure 2A–D). Additionally, an ECAR assessment verified that *HS3ST1* mRNA knockdown decreased ECAR, while *HS3ST1* overexpression elevated ECAR (Figure 2E–H). These findings suggest that HS3ST1 promotes glycolysis in LUAD cells in vitro.

### 4.2. GPC4 Promotes HS3ST1-Mediated Glycolysis in LUAD Cells

**Figure 3:**
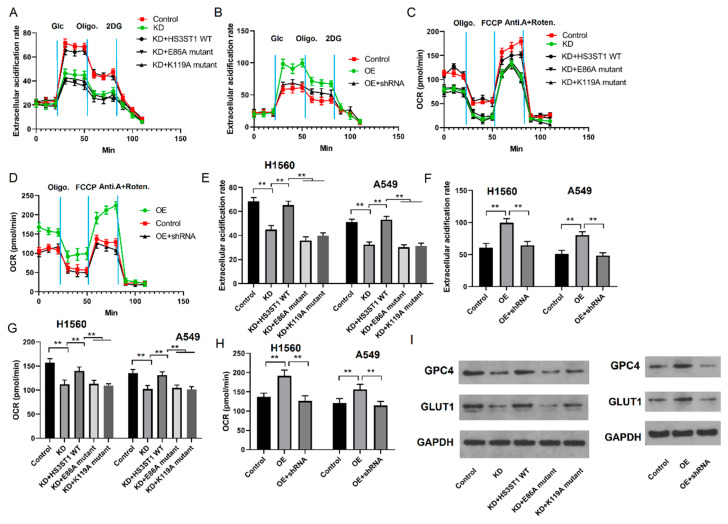
GPC4 enhances HS3ST1-mediated glycolysis in LUAD cells: (**A**–**H**) The ECAR and OCR values in different groups of LUAD A-549 cells. (**I**) The levels of GPC4 and GLUT1 (a glycolytic gene) proteins in various groups of LUAD A-549 cells. Error bars represent the means ± SEM of 3 independent biological experiments. ** *p* < 0.01. OCR is oxygen consumption rate.

Given that HS3ST1 interacts with GPC4 in LUAD cells, we explored whether GPC4 also regulates glycolysis in LUAD cell lines via HS3ST1. We introduced wild-type (WT) *GPC4* and bundling-defective mutants, E86A and K119A, into *GPC4* knockdown cells. *GPC4* knockdown reduced ECAR and OCR in the H1560 and A-549 cell lines (Figure 3A–H), confirming that GPC4 enhances glycolysis in LUAD cells. Moreover, ectopically expressed WT *HS3ST1*, but not the bundling-defective mutants, partially restored ECAR and OCR in the *GPC4*-knockdown LUAD cell lines. Furthermore, we conducted Western blotting to assess the expression of solute carrier family 2 (GLUT1) protein, a key glucose transporter in mammalian cells. We found that the GLUT1 protein levels substantially decreased following *GPC4* knockdown, whereas they were elevated after *GPC4* overexpression (Figure 3I). Therefore, our data show that WT *HS3ST1* substantially mitigated the impact of GPC4 on glycolysis. These results also strongly support the notion that GPC4 promotes HS3ST1-mediated glycolysis in LUAD in vitro.

### 4.3. Identification of lncRNA OIP5-AS1 in Hypoxic LUAD Cells

**Figure 4:**
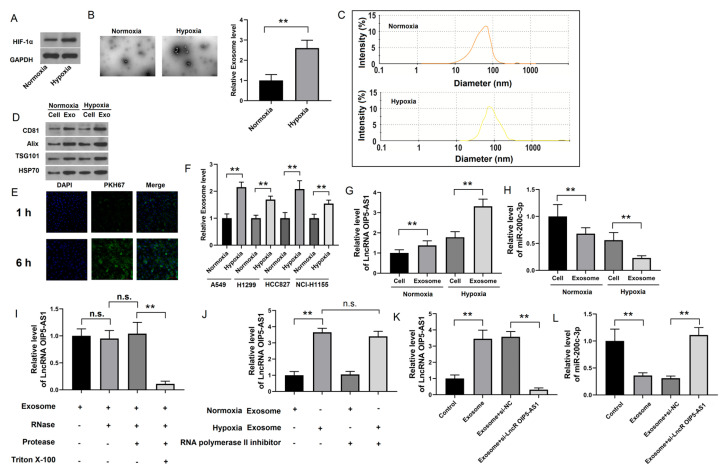
Identification of lncRNA OIP5-AS1 in hypoxic LUAD cells: (**A**) The levels of HIF-1α protein in normoxic and hypoxic LUAD A-549 cells. (**B**,**C**) Exosomes derived from normoxic or hypoxic LUAD A-549 cells were identified by TEM and NTA. (**D**) The protein expression of exosome markers TSG101, Alix, HSP70, and CD81 in normoxic and hypoxic LUAD A-549 cells. (**E**) Immunofluorescence of PKH67 in normoxic and hypoxic LUAD A-549 cells. (**F**) The exosome-level comparison between normoxic and hypoxic LUAD cell lines. (**G**,**H**) The relative expression of lncRNA OIP5-AS1 and miR-200c-3p in normoxic and hypoxic LUAD A-549 cells. (**I**–**L**) The relative expression of lncRNA OIP5-AS1 or miR-200c-3p in different groups of normoxic or hypoxic LUAD A-549 cells. Error bars represent the means ± SEM of 3 independent biological experiments. ** *p* < 0.01. n.s., not significant.

As hypoxia is a prominent characteristic of LUAD, we cultured LUAD cells under hypoxic (1% O_2_) and normoxic (20% O_2_) conditions to assess HIF-1α expression. We verified that its expression was higher in hypoxic cells than that in normoxic cells (Figure 4A), confirming hypoxia in our cell lines. Next, we identified exosomes derived from normoxic and hypoxic LUAD cells to investigate whether hypoxia-derived exosomes influence the malignancy of LUAD. The hypoxia-derived exosomes were typical round particles, 100 nm in diameter (Figure 4B,C). A Western blotting assay showed that they also exhibited a substantial upregulation of exosome markers, such as tumor susceptibility 101 (TSG101), ALG-2-interacting protein X (Alix), heat shock 70kDa protein 1A (HSP70), and CD81 (Figure 4D), indicating enhanced exosome secretion under hypoxia. In addition, this outcome was consistent across the various LUAD cell lines (Figure 4F). Immunofluorescence indicated that co-culturing exosomes from hypoxic LUAD cells with normoxic cells caused the PKH67 exosome-labeling dye to co-localize with DAPI, suggesting that exosomes from hypoxic LUAD cells fuse with normoxic LUAD cells in vitro (Figure 4E). These findings demonstrated that hypoxia increases exosome secretion from hypoxic LUAD cells and exosome fusion with normoxic cells.

Non-coding RNAs (ncRNAs) are highly abundant in exosomes and play crucial roles in cancer regulation [28]. We performed a PCR assay to assess the expression of the lncRNAs OIP5-AS1 and miR-200c-3p and decipher whether hypoxia-induced exosomal lncRNAs and miRNAs impact tumor progression. While the lncRNA OIP5-AS1 was significantly more abundant in hypoxic LUAD cells than in their paired normoxic cells, miR-200c-3p was downregulated (Figure 4G,H). Proteinase and RNase protection assays also illustrated that the lncRNA OIP5-AS1 specifically resided in the exosome lumen and exhibited RNase resistance (Figure 4I). Furthermore, when A-549 cells were exposed to exosomes from hypoxic LUAD cells, the levels of the lncRNA OIP5-AS1 increased in A-549 cells and remained high even in the presence of an RNA polymerase II inhibitor (Figure 4J). These results show that the internalization of the lncRNA OIP5-AS1 in LUAD cells is mediated by exosomes released from hypoxic cells and not due to self-synthesis by LUAD cells. We also observed that the knockdown of the lncRNA OIP5-AS1 in LUAD cells resulted in exosomes transmitting fewer miR-200c-3p than those transfected with negative control siRNA (si-NC) (Figure 4K,L). In conclusion, these data suggest that hypoxic LUAD cells produce the lncRNA OIP5-AS1 and transfer it to LUAD cells via exosome secretion.

### 4.4. Hypoxia-Derived Exosomal lncRNA OIP5-AS1 Targets HS3ST1-GPC4 and miR-200c-3p in LUAD Cells

**Figure 5:**
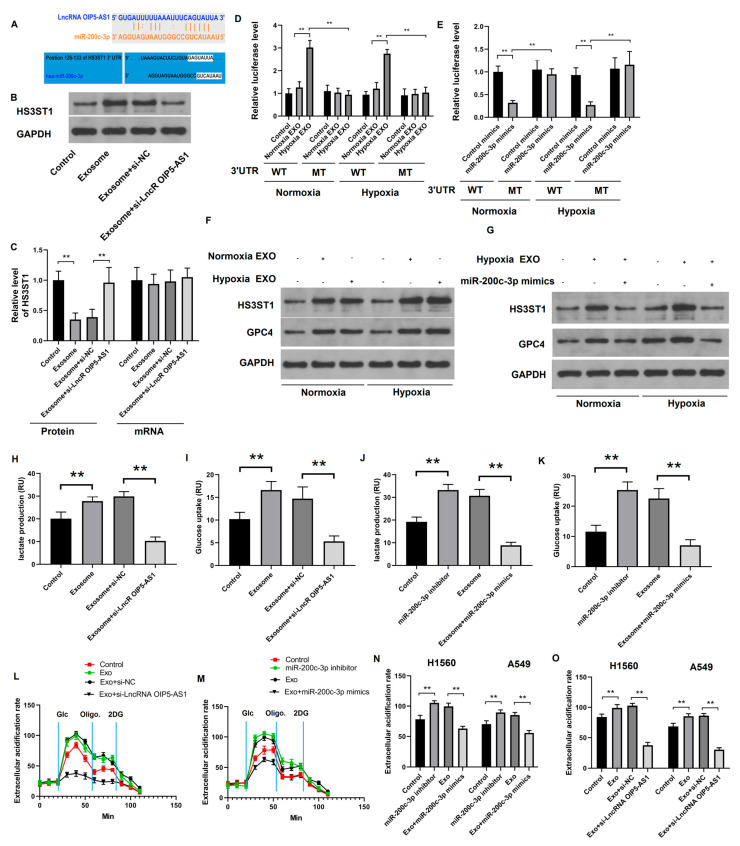
Hypoxia-derived exosomal lncRNA OIP5-AS1 increases glycolysis via miR-200c-3p axis in LUAD cells: (**A**) The potential binding site between lncRNA OIP5-AS1 and miR-200c-3p or between *HS3ST1* mRNA and miR-200c-3p were predicted using the TargetScan dataset. (**B**,**C**) The expression of *HS3ST1* mRNA in different exosome groups. (**D**,**E**) Dual-luciferase reporter assay confirming the direct binding between miR-200c-3p and the *HS3ST1* mRNA 3′ UTR regions. (**F**,**G**) The levels of HS3ST1 or GPC4 protein in LUAD A-549 cells after treatment with different exosome groups. GAPDH was used for normalization. (**H**–**K**) Lactate production and glucose uptake and (**L**–**O**) the ECAR of LUAD A-549 cells after treating them with diverse exosome groups. Error bars represent the means ± SEM of 3 independent biological experiments. ** *p* < 0.01.

Our results indicate that the hypoxia-derived exosomal lncRNA OIP5-AS1 may influence LUAD progression. We used the TargetScan web server to predict the targets of miR-200c-3p and determine the mechanism of action of the lncRNA OIP5-AS1 in LUAD cells. We showed that miR-200c-3p has binding sites on the lncRNA OIP5-AS1 and the *HS3ST1* mRNA (Figure 5A). Subsequently, we isolated exosomes derived from WT or lncRNA OIP5-AS1 knockdown A-549 cells and co-cultured them with LUAD cells. Using Western blotting and RT-qPCR, we assessed the effect of perturbations in the lncRNA OIP5-AS1’s expression on HS3ST1 abundance in co-cultured LUAD cells (Figure 5B,C). Exosomes lacking the lncRNA OIP5-AS1 downregulated HS3ST1 protein expression, although the *HS3ST1* mRNA remained largely unaffected, suggesting that exosomes primarily influenced HS3ST1 protein expression in LUAD cells. We performed a luciferase reporter assay to confirm the direct interaction between miR-200c-3p and the 3′-UTR of *HS3ST1* mRNA. We used luciferase reporter plasmids containing the WT or mutant (MT) *HS3ST1* mRNA 3′ UTR sites harboring the miR-200c-3p binding site. The transfection of cells with miR-200c-3p mimics and exposing the cells to exosomes derived from hypoxic LUAD cells noticeably reduced the luciferase activity compared with cells transfected with control mimics (non-targeting mimics) and cells treated with exosomes from normoxic LUAD cells (Figure 5D,E). As HS3ST1 interacts with GPC4 in LUAD tissues, we assessed the HS3ST1 and GPC4 protein expression in various LUAD cell lines. Hypoxic LUAD cell-derived exosomes lead to increased expression of *HS3ST1* and *GPC4*, especially in hypoxic cells. Treatment with miR-200c-3p mimics inhibited the promotion of hypoxic LUAD cell-derived exosomes in vitro (Figure 5F,G). These findings indicate that the exosomal lncRNA OIP5-AS1, derived from hypoxic LUAD cells, targets HS3ST1-GPC4 and miR-200c-3p.

### 4.5. Hypoxia-Derived Exosomal lncRNA OIP5-AS1 Promotes Glycolysis via miR-200c-3p Axis in LUAD Cells

Lung adenocarcinoma cells were treated with various exosomes and their glycolysis status was assessed. We found that LUAD cell-derived exosomes or an miR-200c-3p inhibitor enhanced lactate production and glucose uptake. However, the knockdown of the lncRNA OIP5-AS1 in exosomes or their transfection with miR-200c-3p mimics restored the glycolysis effect observed during co-culturing (Figure 5H–K). These results suggest that the lncRNA OIP5-AS1 promotes glycolysis in vitro, a process that is negatively regulated by miR-200c-3p. Measuring ECAR also confirmed that knocking down the lncRNA OIP5-AS1 or transfecting the cells with miR-200c-3p mimics reduced ECAR, while an miR-200c-3p inhibitor increased it (Figure 5L–O). These findings indicate that the exosomal lncRNA OIP5-AS1, derived from hypoxic LUAD cells, promotes glycolysis by regulating miR-200c-3p in LUAD cells.

### 4.6. Hypoxia-Derived Exosomal lncRNA OIP5-AS1 Promotes LUAD Proliferation and Metastasis via miR-200c-3p In Vitro

**Figure 6:**
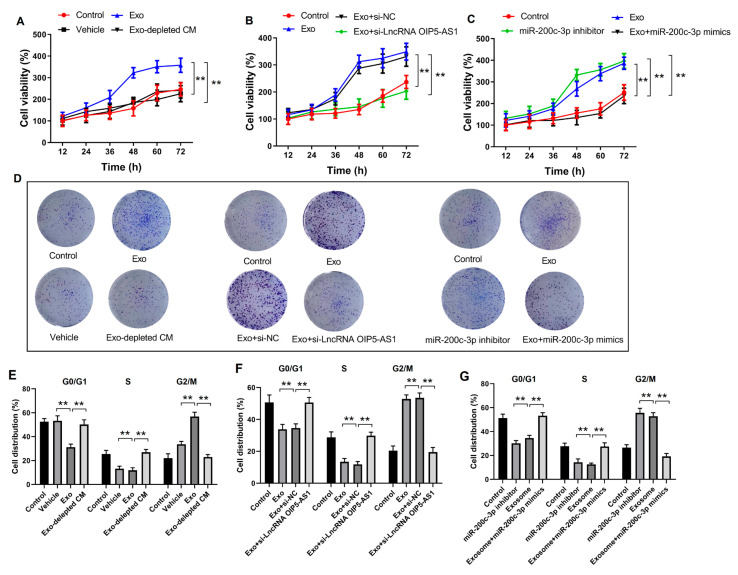
Hypoxia-derived exosomal lncRNA OIP5-AS1 promotes LUAD cell proliferation and metastasis via miR-200c-3p axis in vitro. (**A**–**C**) The cell viability, (**D**) colony formation, (**E**–**G**) cell cycle assay of LUAD A-549 cells after treatment with different exosome groups. Error bars represent the means ± SEM of 3 independent biological experiments. ** *p* < 0.01.

We isolated distinct exosome subgroups from LUAD cells under hypoxic conditions. We co-cultured these cells with different LUAD cell lines to determine the significance of the hypoxia-derived exosomal lncRNA OIP5-AS1 in LUAD. Our investigations encompassed colony formation, cell viability and cell cycle, all designed to evaluate the effect of hypoxia-induced exosomes on in vitro cell proliferation and migration. The treatment of LUAD cells with hypoxic exosomes resulted in increased cell viability, higher proportions of cells in the G2/M phase (Figure 6A,E). The representative images of cell cycle results measured by flow cytometry are shown in Supplementary Figure S2. Remarkably, when hypoxia-derived exosomes were removed from the culture medium, the effects of viability and proliferation on the co-cultured cells were reversed. These results suggested that hypoxia-derived exosomes promote cell proliferation in vitro. Nevertheless, this effect was abolished by knocking down the lncRNA OIP5-AS1 in exosomes or transfecting them with miR-200c-3p mimics (Figure 6B,C,F,G). Additionally, colony formation assays showed that LUAD cell migration significantly increased following treatment with hypoxia-derived exosomes compared to treatment with a hypoxia-derived exosome-depleted culture medium (Figure 6D). Conversely, LUAD cell migration was restored after treating exosomes with si-lncRNA OIP5-AS1 or transfecting them with miR-200c-3p mimics. These results indicate that the hypoxia-derived exosomal lncRNA OIP5-AS1 promotes LUAD proliferation and metastasis in vitro by targeting miR-200c-3p.

### 4.7. Hypoxia-Derived Exosomal lncRNA OIP5-AS1 Promotes LUAD Tumor Growth and Metastasis via miR-200c-3p Axis In Vivo

**Figure 7:**
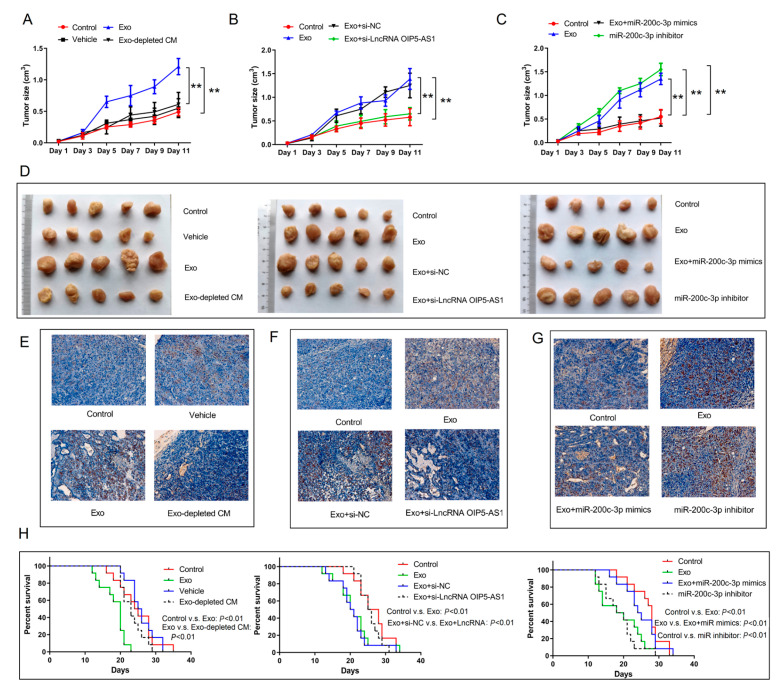
Hypoxia-derived exosomal lncRNA OIP5-AS1 intensifies LUAD tumor growth and metastasis via miR-200c-3p axis in vivo. (**A**–**D**) The tumor size of xenograft tumor samples in mouse models after treatment with different exosome groups. (**E**–**G**) Hematoxylin–eosin staining of LUAD metastasis nodules. (**H**) The percent survival of xenograft tumor mice after treatment with different exosome groups. Error bars represent the means ± SEM of 3 independent biological experiments. ** *p* < 0.01.

We established a mouse xenograft tumor model using A-549 cells to explore the malignant effects of the exosomal lncRNA OIP5-AS1 in vivo. The mice received intravenous injections of various exosome subgroups every 3 days. After six administrations, on day 30, the mice were euthanized and the tumors were collected. As anticipated, hypoxia-derived exosomes augmented the xenograft tumor size and weight. Conversely, hypoxia-depleted exosomes or exosomes containing si-lncRNA OIP5-AS1 counteracted the accelerated tumor growth triggered by hypoxia-derived exosomes (Figure 7A,B,D). The miR-200c-3p inhibitor also mitigated xenograft tumor growth induced by hypoxia-derived exosomes in vivo (Figure 7C,D). In addition, the hematoxylin–eosin staining of LUAD metastatic nodules produced similar results (Figure 7E–G). Moreover, injecting hypoxia-derived exosomes reduced the survival percentage of the xenograft tumor mice, and this effect was reversed when hypoxia-derived exosomes were depleted or the exosomes contained si-lncRNA OIP5-AS1i or an miR-200c-3p inhibitor (Figure 7H). These in vivo findings support our in vitro experiments, emphasizing that the hypoxia-derived exosomal lncRNA OIP5-AS1 promotes LUAD tumor growth and metastasis via miR-200c-3p.

### 4.8. Long Non-Coding RNA OIP5-AS1 Is Upregulated in LUAD Tissues, Serum, and Serum Exosomes

**Figure 8:**
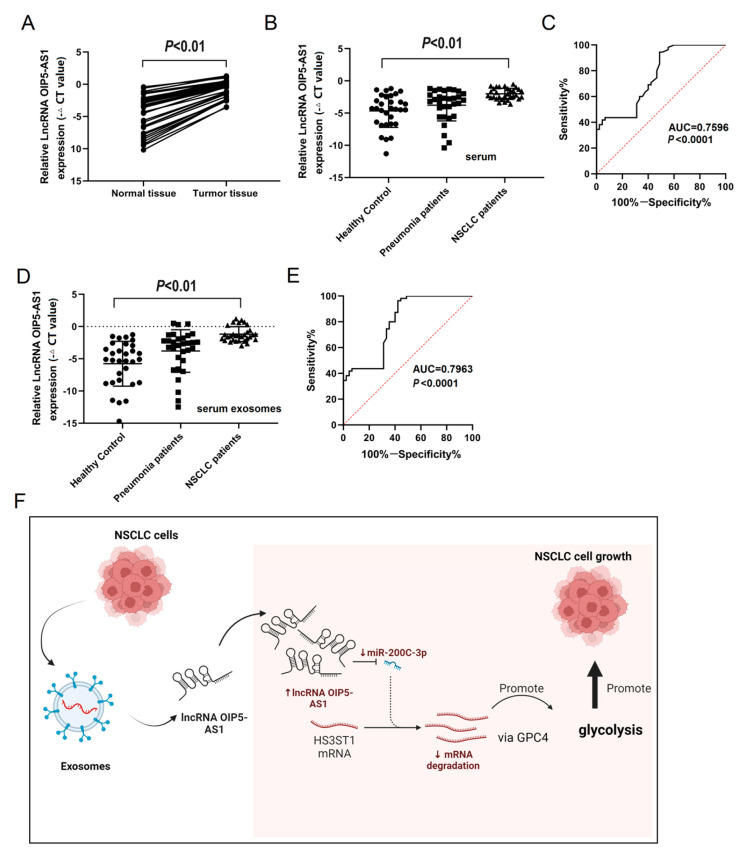
Long non-coding RNA OIP5-AS1 expression is upregulated in human LUAD tissues, serum, and serum exosomes: (**A**) The relative expression of lncRNA OIP5-AS1 in LUAD tissues. (**B**) The relative expression of lncRNA OIP5-AS1 in the serum of patients with NSCLC patients. (**C**) The ROC curve of exosomes derived from serum samples. (**D**) The relative expression of exosomal lncRNA OIP5-AS1 in the serum of patients with NSCLC. (**E**) The ROC curve of lncRNA OIP5-AS1 derived from serum exosomes. (**F**) The summary of the mechanism discovered in the study. Error bars represent the means ± SEM of 3 independent biological experiments. ROC, receiver operating characteristic.

We gathered 31 pairs of human LUAD tissues and their corresponding adjacent normal tissues to assess the expression of the lncRNA OIP5-AS1 using RT-qPCR. The findings unambiguously confirmed the significantly upregulated expression of the lncRNA OIP5-AS1 in LUAD tissues versus adjacent normal tissues (Figure 8A). Furthermore, we examined the expression of the lncRNA OIP5-AS1 in serum samples from various individuals. A noticeable increase in lncRNA OIP5-AS1 expression was detected in the serum samples of patients with NSCLC compared to patients with pneumonia or healthy donors (Figure 8B). Finally, we calculated receiver operating characteristic (ROC) curves to estimate the diagnostic potential of serum exosomes as LUAD biomarkers, yielding an area under the curve (AUC) of 0.7596 (Figure 8C). Additionally, we determined the lncRNA OIP5-AS1 levels in exosomes derived from serum samples, revealing markedly higher transcript levels in patients with NSCLC than in those with pneumonia or healthy donors, as well as an AUC of 0.7963 (Figure 8D,E). In conclusion, these analyses robustly demonstrated significant lncRNA OIP5-AS1 overexpression in LUAD tissues, serum, and serum exosomes.

## 5. Discussion

Lung carcinoma (LC) is a serious health issue and remains a leading cause of cancer-related fatalities worldwide [29]. Pathologically, lung cancer is broadly classified into two primary types: small-cell lung carcinoma (SCLC) and NSCLC, with NSCLC being the dominant subtype [30]. Within the NSCLC category, LUAD is the most common histological variant, accounting for approximately 40% of the cases [31]. A notable focus has been placed on early surgical interventions for LUAD, leading to enhanced survival rates for individuals diagnosed at an initial disease stage [32]. However, despite advancements in lung cancer research, LUAD remains an aggressive malignant neoplasm with related risks and a continued adverse influence on patient survival and prognosis. Although targeted therapies based on specific biomarkers show promise, their broader application requires further investigation [33], demanding an accelerated search for novel LUAD-related biomarkers.

Hypoxia is a distinguishing feature of solid tumors and plays a central role in glycolysis, cell proliferation, and migration [34,35,36]. An increasing body of evidence indicates that hypoxia within tumor tissues influences the tumor immune microenvironment, fostering immune evasion and immunosuppression [37]. Hypoxia also suppresses the activation and effectiveness of CD8^+^ T cells, which are associated with more favorable outcomes in various cancers and disrupts the function of natural killer (NK) cells [38,39]. Moreover, it drives metabolic reprogramming to meet the energy demands of tumor cells during acute hypoxic stress, particularly by promoting glycolysis at the transcriptional level [40,41]. Because reported hypoxia-specific biomarkers of the LUAD environment are scarce, identifying hypoxia-related genes in LUAD for developing prognostic signatures is of great practical importance. In this study, we demonstrated that hypoxia-derived exosomes can enhance glycolysis in LUAD cells and promote their proliferation and metastasis in vitro. We also showed that hypoxia-derived exosomes stimulate LUAD tumor growth and metastasis in vivo. Therefore, we demonstrated a substantial role of hypoxia-derived exosomes in the mechanisms that promote LUAD progression.

Elevated glycolysis, characterized by an increased glucose uptake and lactate production, is a hallmark of cancer metabolism [42]. This heightened glycolysis is an adaptation of cancer cells to the hypoxic tumor microenvironment, providing a continuous energy source for fuel cell proliferation, invasion, and migration [43,44]. Therefore, the exploration of glycolysis as a potential therapeutic approach for cancer has gained much attention [45]. Recent research has revealed a connection between glycolysis and tumor initiation and progression, underscoring its pivotal role in driving LUAD advancement [46,47,48]. However, the regulation of hypoxic stress and its connection to glycolysis in LUAD remains relatively unexplored. In this study, we investigated the regulatory and functional aspects of hypoxia-induced glycolysis using cell-based experiments, mouse models, and human LUAD cohorts. We found that GPC4 may facilitate HS3ST1-mediated glycolysis in LUAD cells. Additionally, we observed that the hypoxia-derived exosomal lncRNA OIP5-AS1 has the potential to promote glycolysis in LUAD cells, an effect mediated through the miR-200c-3p axis. These findings underscore the importance of glycolysis, particularly hypoxia-mediated glycolysis, in LUAD pathogenesis and offer new insights into the clinical prevention and treatment of the disease.

Increasing evidence suggests that exosomes are crucial mediators in the communication between different cell types, carrying complex biological information such as mRNAs, non-coding RNAs, and soluble and transmembrane proteins [49,50]. These exosomes can disseminate to nearby cells or be transported to remote locations, serving as carriers of information to specific recipient cells [51,52]. Exosomes derived from cancer cells play a significant role in tumor initiation and progression [53,54]. Similarly, lncRNAs, a subset of non-coding RNAs > 200 nucleotides in length, participate in the mechanisms of diverse cancers [55,56,57,58]. Remarkably, lncRNAs control the expression of target genes at multiple levels, including chromatin remodeling, splicing, transcriptional regulation, and post-transcriptional modifications. Consequently, they modulate critical cellular processes including proliferation, apoptosis, and migration [57,58]. Furthermore, lncRNAs influence the expression of various miRNAs and transcription factors, thereby contributing to the regulation of cellular processes [18,59,60,61]. In this study, we explored the potential role of hypoxia-derived exosomes in LUAD cells and xenograft tumor models. Our research indicated that the lncRNA OIP5-AS1 is overexpressed in LUAD tissues, serum, and serum-derived exosomes. We also discovered that the hypoxia-derived exosomal lncRNA OIP5-AS1 targets HS3ST1-GPC4 and miR-200c-3p in LUAD cells. Furthermore, this lncRNA promotes glycolysis, cell proliferation, and metastasis via the miR-200c-3p axis in LUAD cells. These findings suggest that the lncRNA OIP5-AS1 from hypoxia-derived exosomes plays essential roles in LUAD prognosis, pathogenesis, and therapy resistance, potentially leading to novel strategies for LUAD patient care.

Heparan sulfate proteoglycans are integral components of the cell microenvironment and play crucial roles in cell–cell interactions, signal transduction, migration, and adhesion [62]. Evidence shows that these proteins are involved in tumor proliferation, metastasis, and angiogenesis [63]. The HS3ST1 protein is a rate-limiting enzyme that is essential for heparan sulfate synthesis that is overexpressed and associated with various cancers, affecting proliferation and apoptosis [64,65]. However, the effects and underlying mechanisms of LUAD remain unclear. We found that HS3ST1 was overexpressed and interacted with GPC4, thereby facilitating HS3ST1-mediated glycolysis in LUAD cells. Furthermore, the hypoxia-derived exosomal lncRNA OIP5-AS1 binds HS3ST1-GPC4 and miR-200c-3p, promoting glycolysis in LUAD cells. These findings indicate that HS3ST1 may play a role in the pathogenesis of LUAD. 

## 6. Conclusions

In conclusion, our study underscores the potential role of the hypoxia-derived exosomal lncRNA OIP5-AS1 in LUAD. We found that GPC4 promotes HS3ST1-mediated glycolysis, and the hypoxia-derived exosomal lncRNA OIP5-AS1 promotes glycolysis via the miR-200c-3p axis in LUAD cells. The hypoxia-derived exosomal lncRNA OIP5-AS1 enhances LUAD cell proliferation and metastasis in vitro and promotes LUAD tumor growth and metastasis via miR-200c-3p in vivo. These findings highlight that the hypoxia-derived exosomal lncRNA OIP5-AS1 may participate in LUAD progression.

## Data Availability

The datasets generated and/or analyzed during the current study are available from the corresponding author upon reasonable request.

## Supplementary Materials

The following supporting information can be downloaded at https://www.mdpi.com/article/10.3390/cancers16040695/s1: Figure S1: The uncropped blots and molecular weight markers; Figure S2: The representative images of cell cycle results measured by flow cytometry; Table S1: Antibodies used in the study; Table S2: Primers used in the study.
