# Supplementary material for: Hypoxia-Derived Exosomes Promote Lung Adenocarcinoma by Regulating HS3ST1-GPC4-Mediated Glycolysis
Source: Cancers (Basel). 2024 Feb 6;16(4):695. doi: 10.3390/cancers16040695 (PMC10886556; doi:10.3390/cancers16040695)
Supplement: Supplementary file 1 [file cancers-16-00695-s001.zip › Table S1.pdf]

Supplementary Table S1. Antibodies used in the study.

Reagent	Source	Identifier
Primary antibodies		
Anti-HS3ST1 antibody, rabbit monoclonal	Abcam	ab252833
Anti-GPC4 antibody, rabbit monoclonal	Abcam	ab197896
Anti-GLUT1 antibody, rabbit polyclonal	Abcam	ab237704
Anti-CD81 antibody, rabbit monoclonal	Abcam	ab308304
Anti-Alix antibody, rabbit monoclonal	Cell Signaling Technology	#3771
Anti-TSG101 antibody, rabbit monoclonal	Cell Signaling Technology	#3230
Anti-HSP70 antibody, rabbit polyclonal	Cell Signaling Technology	#9145
Anti-GAPDH antibody, rabbit polyclonal	Abcam	ab9485
Secondary antibodies		
Goat Anti-Rabbit IgG H&L (HRP)	Abcam	ab97051
