# Supplementary material for: Hypoxia-Derived Exosomes Promote Lung Adenocarcinoma by Regulating HS3ST1-GPC4-Mediated Glycolysis
Source: Cancers (Basel). 2024 Feb 6;16(4):695. doi: 10.3390/cancers16040695 (PMC10886556; doi:10.3390/cancers16040695)
Supplement: Supplementary file 1 [file cancers-16-00695-s001.zip › Table S2.pdf]

Supplementary Table S2. Primers used in the study.

RT-PCR Primers		
HS3ST1	GGAAAGAACACCCGGAAATGA	GTGCTGCAACTGGGTAAACAC
LncRNA OIP5-AS1	TCACCAAGTTTGGACACCGT	ATAGTGGGGCAGGTCCTTCT
miR-200c-3p	GTAGTGGTGGCCTCTTGGAC	GAGGAGTGGGTGTTGGGAGA
GAPDH	AGTGTGACGTGGACATCCGCA	ATCCACATCTGCTGGAAGGTGGAC
