# Supplementary figures and images for: Hypoxia-Derived Exosomes Promote Lung Adenocarcinoma by Regulating HS3ST1-GPC4-Mediated Glycolysis
Source: Cancers (Basel). 2024 Feb 6;16(4):695. doi: 10.3390/cancers16040695 (PMC10886556; doi:10.3390/cancers16040695)

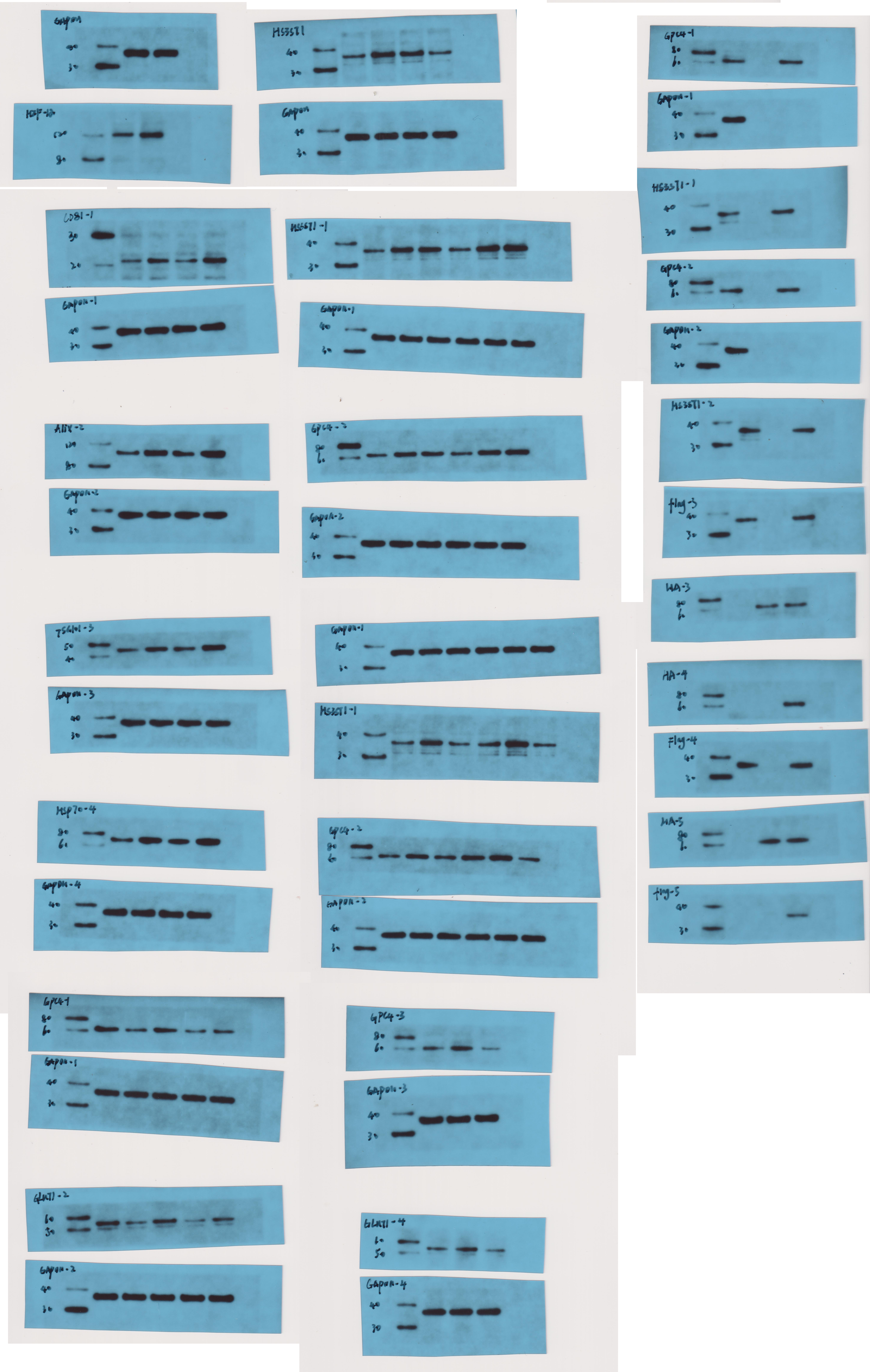

Supplement: Supplementary file 1 [file cancers-16-00695-s001.zip › Figure S1.tif]

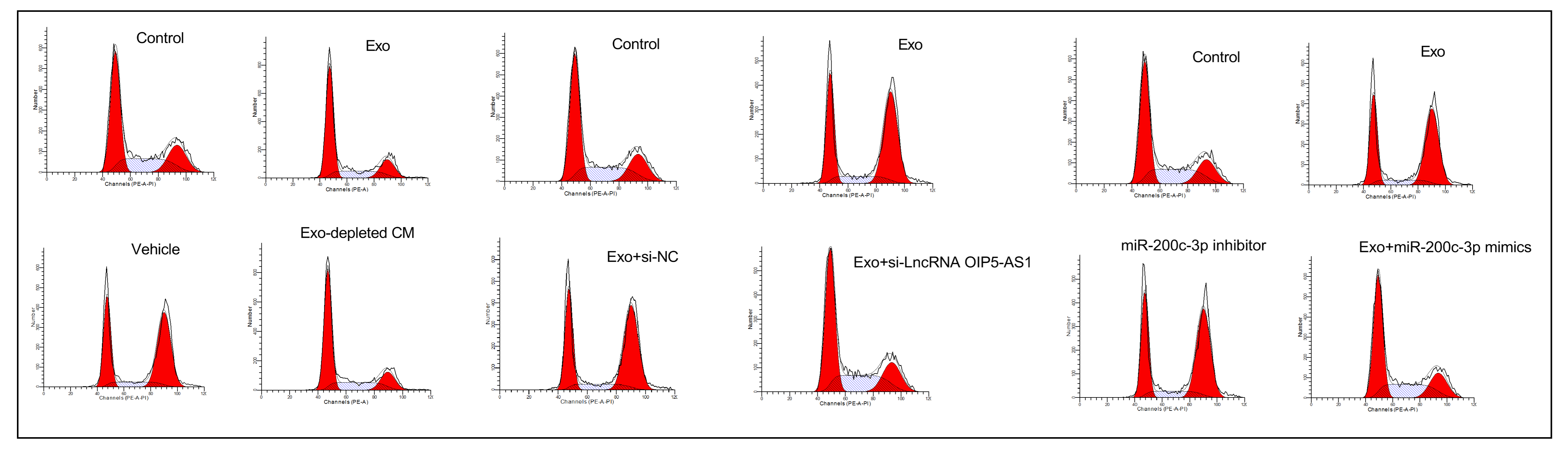

Supplement: Supplementary file 1 [file cancers-16-00695-s001.zip › Figure S2.tif]
